# *In Vivo* Ultrasound Molecular Imaging of SDF-1 Expression in a Swine Model of Acute Myocardial Infarction

**DOI:** 10.3389/fphar.2019.00899

**Published:** 2019-08-21

**Authors:** Meng Wang, Rong Hu, Yuanyuan Yang, Liping Xiang, Yuming Mu

**Affiliations:** Department of Echocardiography, First Affiliated Hospital, Xinjiang Medical University, Ürümqi, China

**Keywords:** SDF-1, targeted microbubbles, ultrasound molecular imaging, acute myocardial infarction, in vivo

## Abstract

**Background:** Stem cell therapy of acute myocardial infarction (AMI) is proving to be a promising approach to repair the injured myocardia. The time window for stem cell transplantation is crucial yet difficult to determine since it produces different therapeutic effects at different times after myocardial infarction. Stromal cell-derived factor-1 (SDF- 1) plays a pivotal role in the mobilization, homing, proliferation, and differentiation of transplanted stem cells. Here, by using ultrasound molecular imaging *via* targeted microbubbles, we determined the dynamic expression of SDF-1 in a swine model of AMI *in vivo*.

**Methods:** Twenty-four miniswine were randomly selected for the control group and the AMI model group, which underwent ligation of the left anterior descending coronary artery (LAD). The AMI animals were randomly divided into six experimental groups according to the duration of the myocardial infarction. All animals were subjected to ultrasound molecular imaging through injections with targeted microbubbles (T + T group) or nontargeted control microbubbles (T + C group). The values of the myocardial perfusion parameters (A, β, and A × β) were determined using Q-Lab (Philips ultrasound, version 9.0), and the expression level of SDF-1 was analyzed by real-time polymerase chain reaction (RT-PCR).

**Results:** Our results showed that the expression of SDF-1 gradually increased and peaked at 1 week after AMI. The trend is well reflected by ultrasound molecular imaging in the myocardial perfusion parameters. The A, β, and A × β values correlated with SDF-1 in the T + T group (*r* = 0.887, 0.892, and 0.942; *P* < 0.05). Regression equations were established for the relationships of the A, β, and A × β values (*X*) with SDF-1 (*Y*): *Y* = 0.699*X* − 0.6048, *Y* = 0.4698*X* + 0.3282, and *Y* = 0.0945*X* + 0.6685, respectively (*R* ^2^ = 0.772, 0.7957, and 0.8871; *P* < 0.05).

**Conclusions:** Our finding demonstrated that ultrasound molecular imaging could be used to evaluate the expression dynamics of SDF-1 after AMI.

## Introduction

Acute myocardial infarction (AMI), which is defined as myocardial cell death caused by prolonged ischemia, is one of the leading causes of cardiovascular disease mortality worldwide (Mozaffarian et al., 2016). Although various cardiovascular therapies, such as percutaneous coronary intervention, implantable defibrillators, and pharmacotherapeutics, have improved heart functions after AMI over recent decades, these treatments do not actively restore or regenerate the damaged myocardial tissue (Pfeffer and Braunwald, 1990; Kuraitis et al., 2011; Roger et al., 2012). The 1-year mortality rate (13%) after an AMI and the resultant left ventricular (LV) systolic dysfunction remain high (Pfeffer et al., 2003).

Recently, replenishing lost cardiomyocytes using stem cell therapy was recognized to be an ideal strategy to retain cardiac function and prevent heart failure (Naaijkens et al., 2014). Several stem cells isolated from different tissue sources have been successfully utilized in therapy for post-AMI in preclinical and clinical studies. The first stem cells used were adult mesenchymal stem cells derived from bone marrow (Friedenstein et al., 1974). Zuk et al. showed that adipose-derived stem cells (ASCs), adult mesenchymal stem cells derived from adipose tissue, also have satisfactory effects (Zuk et al., 2002). There is evidence to demonstrate that both stem cell types can differentiate toward cardiomyocytes and endothelial cells and can be home to damaged tissues *in vivo* (Oswald et al., 2004; Pittenger and Martin, 2004; Boyle et al., 2010; Naaijkens et al., 2012). These adult mesenchymal stem cells may secrete growth factors and cytokines that can stimulate cardiovascular repair through a paracrine effect. Although stem cell-based treatments have shown great promise in the treatment of AMI, stem cell transplantation has different effects at different times after myocardial infarction (Friedenstein et al., 1974; Zuk et al., 2002; Oswald et al., 2004; Pittenger and Martin, 2004; Boyle et al., 2010; Naaijkens et al., 2012). Therefore, determining the optimal time at which stem cell transplantation should be performed is critical.

Stromal cell-derived factor-1 (SDF-1), a chemokine protein known as C-X-C motif chemokine 12 (CXCL12), has been shown to be involved in the mobilization, homing, proliferation, and differentiation of stem cells, with a particularly pivotal role in the homing of stem cells to injured myocardium (Huber et al., 2011; MacArthur et al., 2013; Schuh et al., 2014; Won et al., 2014). Studies have also revealed that the expression of SDF-1 differs throughout the acute period after myocardial injury (Boyle et al., 2011; Ghadge et al., 2011). Current techniques for evaluating SDF-1 expression involve *in vitro* investigations of tissue biopsies, which are complex and time-consuming and have limited clinical practicality, reflecting a difficult issue to address in a clinical setting. Fortunately, ultrasound molecular imaging provides another feasible approach to evaluate SDF-1 in local tissue after AMI. Targeted microbubbles can uniquely bind to SDF-1 molecules and allow imaging with a commercial ultrasound transducer in real time and in a noninvasive manner. Therefore, in this study, we attempted to develop a noninvasive detection technique to evaluate SDF-1 expression through ultrasound molecular imaging *in vivo*.

## Materials and Methods

### Animal Grouping

Eight-month-old Chinese miniswine (27 ± 3 kg, both males and females) were obtained from the Laboratorial Animal Center of the First Affiliated Hospital of Xinjiang Medical University. All animals were vaccinated and dewormed, and they exhibited no signs of disease upon clinical examination before surgery. Three of the 24 miniswine were randomly selected as the control group (n = 3, sham operation group: the chest was opened, but ligation of the coronary artery was not performed), and the remaining 21 miniswine underwent ligation of the first diagonal branch of the left anterior descending coronary artery (LAD). Three animals died due to ventricular fibrillation within 50 min before the thoracic cavity was closed. Therefore, the remaining 18 miniswine that survived following LAD ligation were randomly divided into six experimental groups (n = 3): the six groups were assigned based on six different time points (1 day, 3 days, 1 week, 2 weeks, 3 weeks, and 4 weeks) after AMI. All animals were injected with the targeted microbubble ultrasound contrast agent (T + T group) or the nontargeted ultrasound contrast agent (T + C group).

## AMI Animal Model

### Establishment of the AMI Model

The miniswine were anesthetized using ketamine (10–15 mg/kg, intramuscularly) and atropine (25 μg/kg, intramuscularly), and the venous channels of the marginal ear vein were opened using a method published by Yang and Song (Song et al., 2012; Yang et al., 2013). During the operation, the swine were given intravenous anesthetics (ketamine hydrochloride and midazolam, 4.0 mg/kg bw). Mechanical respiratory ventilation was achieved *via* tracheal intubation after the administration of a suxamethonium chloride injection (0.2 mg/kg). In some cases, even after defibrillation, ventricular tachycardia or ventricular fibrillation may occur; therefore, the electrocardiogram, respiration, heart rate, and oxygen saturation were monitored using an electrocardiogram (ECG, USA, HP Cm XL+) monitor during the operation and were recorded every 15 min. An extra defibrillation instrument was available as a precaution. Sublingual pieces of Betaloc 3 (mixed in saline solution after being ground into powder) were applied after anesthesia was induced, and intravenous lidocaine (at an infusion rate of 50 μg/kg per minute) was used to prevent ventricular fibrillation and to maintain the heart rate at 60 to 100 bpm. Consistent with aseptic surgical processes, the chest was opened through the fourth intercostal space along the left border of the sternum. The pericardium was cut, and the heart was exposed. Proper ischemic conditioning was established through two-step ligation (Mu et al., 2009). Briefly, the LAD was ligated with a prolene suture just distal to the first diagonal branch. The slipknot was released after 50 min. The thoracic cavity was closed layer by layer, and ECG monitoring was continued for 30 min after the operation. If ventricular fibrillation occurred, emergency treatment, such as electric defibrillation, intrathoracic pressure, or injection of lidocaine and adrenaline, was implemented; these cardioversion attempts were terminated if they were unsuccessful after more than 30 min. All animals received analgesics (buprenorphine, 0.3 mg) and antimicrobial therapy (penicillin, 2400 IU) twice daily for 3 days after the operation. All animals were subjected to ECG monitoring and echocardiography in the preoperative and postoperative periods, and serum myocardial enzymes were monitored during the 24-h postoperative period.

### Confirmation of the AMI Model

**Figure 1:**
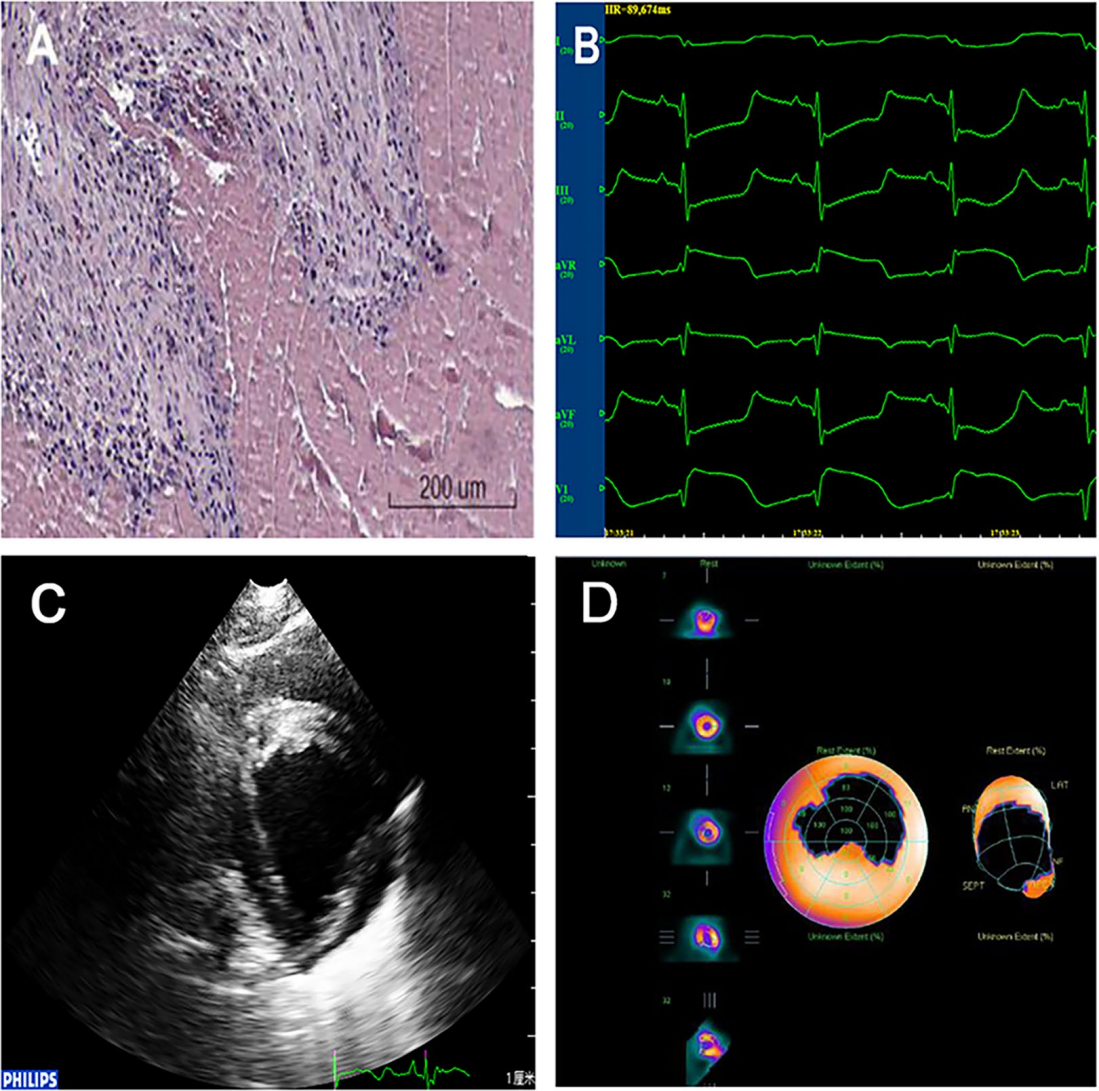
Confirmation of the AMI model. **(A)** *Pathology:* myocardial fiber findings included congestion, edema, neutrophil infiltration, coagulation necrosis, nucleus degeneration, and organization. **(B)** *ECG:* ST segments increased by >0.5 mV in the chest leads. ECG, electrocardiogram; AMI, acute myocardial infarction. **(C)** Ultrasonic cardiograms: Segmental weakening of left ventricular wall motion on two-dimensional echocardiography. **(D)** ECT: Local defects and/or decreases were observed in the radioactive signal.

Establishment of the AMI model was confirmed *via* three methods as follows: 1) *ECG:* The R wave amplitude was significantly increased in the limb lead, ST segments increased by > 0.5 mV in the chest leads for more than 0.5 h, and abnormal Q waves appeared 4 h later. 2) *Emission computed tomography* (ECT, USA, GE, INNOVA 2000): local defects and/or decreases were observed in the radioactive signal. 3) *Pathology:* under light microscopy, several findings related to the myocardial fibers were observed (congestion, edema, neutrophil infiltration, coagulation necrosis, nucleus degeneration, and disorganization). In addition, ultrasonic cardiograms and the levels of the spectrum of serum myocardial enzymes were evaluated according to a previous report (**Figure 1**, **Table S3**) (Munz et al., 2011). The degree of AMI was assessed by gross specimens and ECT. There was no statistically significant difference in the degree of myocardial infarction in each group (**Table S1** and **S2**).

### Preparation of Targeted Microbubbles

Targestar SA (Targeson, Inc., San Diego, CA) is a targetable contrast agent with a streptavidin-coated surface to allow one-step coupling of biotinylated ligands that are suitable for molecular ultrasound imaging. Targeted microbubbles that specifically bind with SDF-1 were prepared according to the manufacturer guidelines. In brief, streptavidin-coated microbubbles were incubated with biotinylated rabbit anti-swine SDF-1 monoclonal antibody (BIOSS, Inc., Beijing, China). The mixture continued to be shaken lightly and was then incubated at room temperature for 20 min and centrifuged at 400 RPM for 4 min to wash out unconjugated antibody (for rapid use within 4 h). Then, the physical-chemical properties and the combined ratio of the prepared targeted ultrasound contrast agent were assessed using a fluorescence microscope and flow cytometer (Decano et al., 2011; Liu et al., 2014; Muramoto et al., 2014). The Targestar SA agent, which was not incubated with any antibody, was used as the nontargeted microbubbles.

### Myocardial Perfusion Parameter Analysis of Myocardial Contrast Echocardiography (MCE)

**Figure 2:**
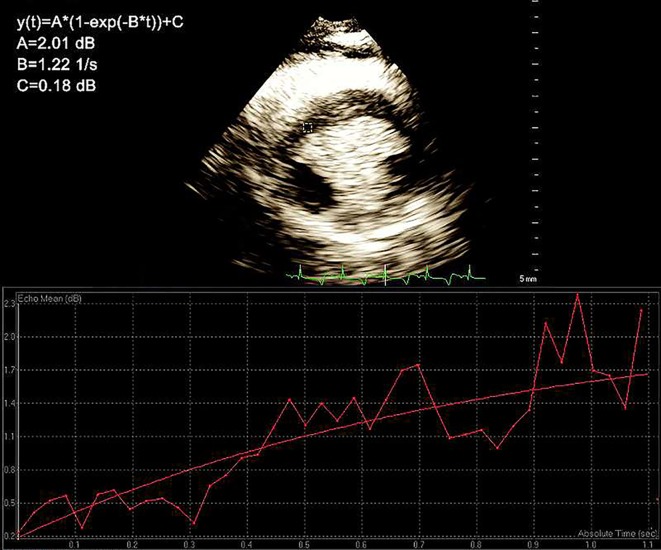
The region of interest (ROI). The values of A (plateau intensity with respect to the myocardial blood volume), β (refill rate with respect to the velocity of myocardial blood flow), and A × β (with respect to myocardial blood flow) as well as the fitting function *Y* = A × (1 − e^-βt^) were determined.

The swine were subjected to anesthesia with ketamine (10–15 mg/kg, intramuscularly) and atropine (25 μg/kg, intramuscularly), and the venous channels of the marginal ear vein were opened. The swine were placed in a lateral position, the limbs were fixed, and the IE-33 ultrasound imaging instrument (cardiac probe model S5-1, 6 Hz; American Philips; probe frequency, 1.8/3.6 MHz; mechanical index, 1.7/0.08) was applied. After satisfactory images were obtained, a contrast agent (0.3 ml/kg) was administered *via* intravenous bolus into the ear vein, followed by 5 ml of saline. After myocardial imaging became stable, scintigraphy was triggered at the parasternal short-axis view of the papillary muscle. A high-mechanical index (1.7) pulse was used to damage myocardial contrast agents. Then, the instrument automatically converted to a low mechanical index state (0.08). To facilitate observation of the contrast agents as they refilled the region of interest (ROI), dynamic images were recorded starting at the time of contrast agent injection and terminating upon the agent’s clearance. All MCE images were analyzed by two experienced doctors using a double-blind Q-Lab workstation (Philips ultrasound, version 9.0). The ROI was set in the perfusion defect at the papillary muscle level. The software automatically recorded the refill time and the intensity curve of the contrast agent. Based on this analysis, the values of A (plateau intensity with respect to the myocardial blood volume), β (refill rate with respect to the velocity of myocardial blood flow), and A × β (with respect to myocardial blood flow) as well as the fitting function *Y* = A × (1 − e^−βt^) were determined (**Figure 2**) (Yang et al., 2013).

### Sampling Methods

After contrast agent analysis and image acquisition, all animals were intravenously premedicated with midazolam hydrochloride and were then anesthetized and euthanized by an intravenous overdose of sodium thiopental and a saturated solution of potassium chloride. The myocardial papillary muscle tissue at the left ventricular short axis was removed, and the infarcted areas in the experimental group animals appeared white; the corresponding myocardial tissue in the control group was also removed.

### Quantitative Real-Time Polymerase Chain Reaction (qRT-PCR)

Myocardial tissues were isolated, dissected, frozen in liquid nitrogen and stored at −80°C until use. Total RNA was extracted using TRIzol reagent (Invitrogen Life Technology, USA) in accordance with the manufacturer’s instructions. The quality of the isolated RNA was evaluated based on the ratio of A260/A280 and gel electrophoresis. Reverse transcription was performed at 37°C for 5 min, at 42°C for 60 min, and at 70°C for 10 min using a PrimeScript RT reagent kit (AMV First Strand cDNA Synthesis Kit, SK2445). To quantify the expression of SDF-1, real-time PCR was performed using a SYBR Green kit (ABI SybrGreen PCR Master Mix) in accordance with the manufacturer’s instructions. The following primers were used: 1) SDF-1 mRNA, forward 5’-ATGCCCTTGCCGATTCTTT-3’ and reverse 5’-TATTGTTGCTCTTCAGCCGTG-3’, with a PCR product of 116 bp, and 2) GAPDH mRNA, forward 5’-ATTTGGCTACAGCAACAGGGT-3’ and reverse 5’-AAGTCAGGAGATGCTCGGTGT-3’, with a PCR product of 172 bp. The conditions for real-time PCR were as follows: denaturation at 95°C for 2 min, followed by 40 cycles of melting at 95°C for 10 s and annealing and extension at 60°C for 40 s. The annealing temperatures for GAPDH and SDF-1 were both 60°C. The data were analyzed using the 2^−ΔΔCT^ method (Lou et al., 2014).

### Reproducibility Analysis

Seven cases were randomly selected to assess intraobserver and interobserver agreement. The intraobserver assessment was performed with >1 week between analyses and with blinding to previous results. Interobserver agreement was assessed between two independent, blinded observers. The intraobserver reproducibility results were expressed as Pearson correlation coefficients. Interobserver reproducibility was determined using Bland–Altman analyses.

### Statistical Analysis

All data are expressed as the means ± standard deviations (SDs). The data were analyzed using SPSS version 17.0 (SPSS, Inc., Chicago, IL). An independent-samples *t*-test was used to compare the RT-PCR results and the myocardial perfusion parameters between the experimental and control groups. The differences in the RT-PCR results for the experimental groups were evaluated using one-way ANOVA. A paired *t*-test was used to compare the myocardial perfusion parameters between the T + T and T + C groups. Pearson correlation was used to analyze the correlations between the myocardial perfusion parameters and SDF-1 in the T + T and T + C groups. A level of *P* < 0.05 was considered statistically significant.

## Results

### Expression Characteristics and RT-PCR Comparison

**Figure 3:**
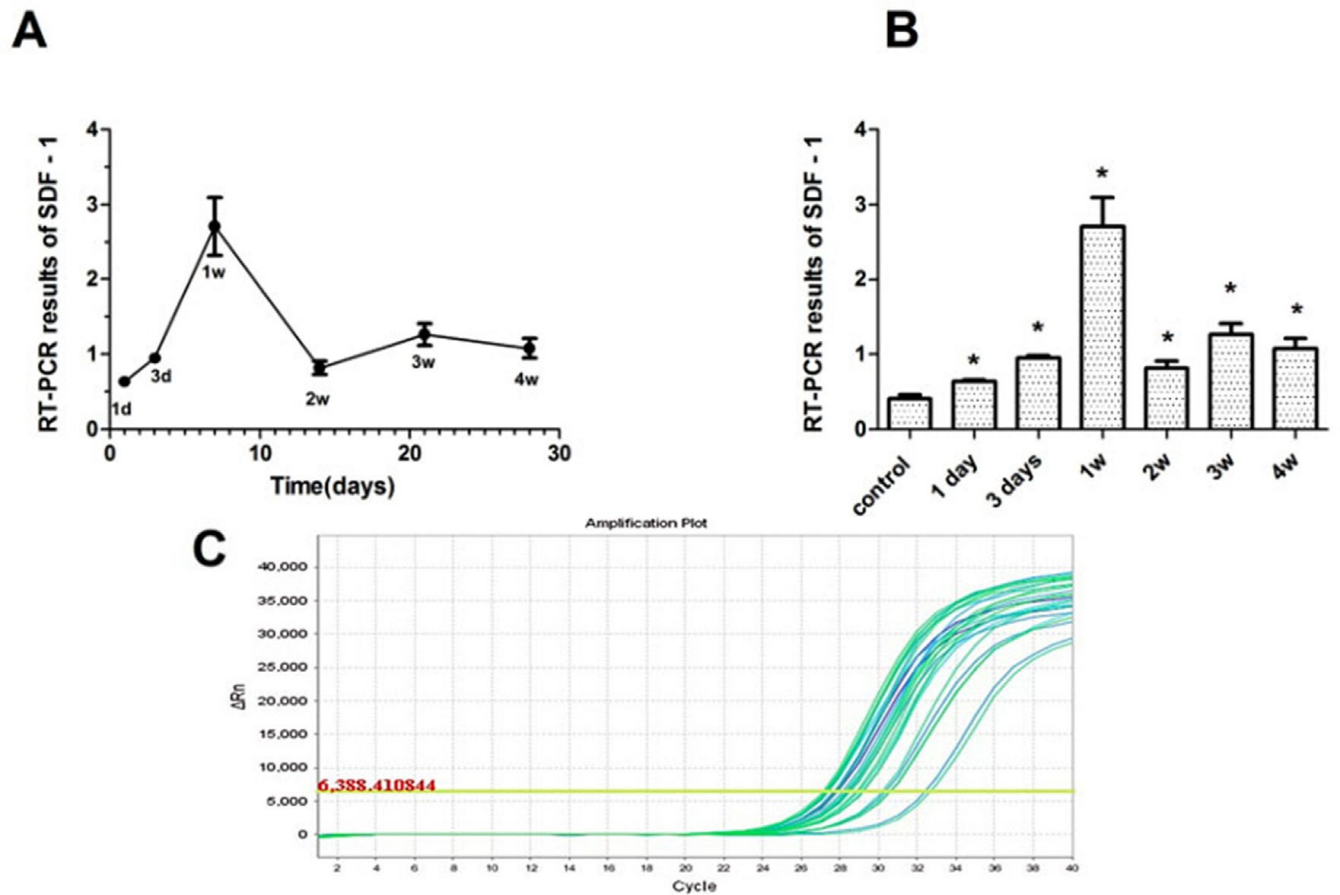
The expression characteristics of SDF-1 in swine myocardium. **(A)** SDF-1 expression at various time points after myocardial infarction; expression peaked at 1 week in the experimental groups. **(B)** SDF-1 expression in the experimental and control groups. The expression of SDF-l in the experimental groups increased more than that in the control group. **(C)** The amplification curve of the SDF-1 gene. *P < 0.05.

The results revealed that SDF-1 was expressed in the AMI experimental groups and the control group. The expression of SDF-l in the experimental groups increased more than that in the control group. After LAD ligation, the expression level of SDF-1 peaked at 1 week in the experimental groups. The differences between the experimental groups and the control group were statistically significant (*P* < 0.05), and the differences between the experimental groups were also statistically significant (*P* < 0.05) (**Table 1** and **Figure 3**).

**Table 1:** The results of SDF-1 RT-PCR (2^−(∆∆Ct^^)^).

Group	Control group	1d	3d	1w	2w	3w	4w
**SDF-1 RT-PCR (n = 3)**	0.4162 ± 0.0737	0.636 ± 0.0505*^ #^	0.949 ± 0.0661* ^#^	2.7079 ± 0.6626*^ #^	0.8200 ± 0.1562*^ #^	1.2641 ± 0.2580*^ #^	1.0793 ± 0.2287*^ #^

Data are presented as the mean ± standard deviation. ^*^P < 0.05 vs. the control group (sham operation). The differences in the experimental groups (different time points after acute myocardial infarction) were statistically significant (^#^P < 0.05).

### Analysis of Myocardial Perfusion Parameters

The myocardial perfusion parameters (A, β, and A × β) of the targeted contrast agent group (T + T group) and the normal contrast agent group (T + C group) were analyzed using Q-Lab software at the parasternal left ventricular short axis of the papillary muscle. The differences between the experimental and control group were statistically significant (*P* < 0.05). All parameters decreased in the experimental groups, and the differences were statistically significant (*P* < 0.05). The differences between the T + T and T + C groups were also statistically significant (*P* < 0.05) (**Table 2**).

**Table 2:** Myocardial perfusion parameter values in the T + T group, T + C group, and control group (A, β, and A × β).

Group		Control group	1d	3d	1w	2w	3w	4w
**T + C group**	A(dB)	6.48 ± 0.39	1.67 ± 0.50*	1.82 ± 0.25*	2.41 ± 1.17*	2.13 ± 0.14*	2.08 ± 0.54*	1.94 ± 0.62*
	β (s^−1^)	6.18 ± 0.21	1.91 ± 0.23*	1.44 ± 0.70*	1.63 ± 0.05*	0.82 ± 0.15*	1.03 ± 0.17*	0.79 ± 0.29*
	A × β(dB/s)	40.13 ± 3.49	3.25 ± 1.35*	2.72 ± 1.48*	3.95 ± 1.93*	1.74 ± 0.37*	2.20 ± 0.92*	1.42 ± 0.15*
**T + T group**	A(dB)	6.41 ± 0.38	2.36 ± 0.24* ^#^	1.96 ± 1.30* ^#^	4.31 ± 1.14* ^#^	2.26 ± 0.26* ^#^	2.18 ± 0.25* ^#^	2.43 ± 0.81*^ #^
	β(澜s^−1^)	6.16 ± 0.16	0.96 ± 0.57* ^#^	1.36 ± 0.37* ^#^	4.85 ± 0.77* ^#^	1.32 ± 0.04* ^#^	1.91 ± 0.29* ^#^	1.28 ± 0.56*^ #^
	A×β(dB/s)	39.55 ± 2.95	2.19 ± 1.06* ^#^	2.69 ± 0.80* ^#^	21.15 ± 7.85* ^#^	3.44 ± 0.31* ^#^	4.19 ± 0.97* ^#^	2.81 ± 0.07* ^#^

Data are presented as the mean ± standard deviation. *P < 0.05 vs. the control group (sham operation). The differences between the T + T group and the T + C group were statistically significant (^#^P < 0.05). T + T: targeted microbubbles ultrasound contrast agent. T + C: normal ultrasound contrast agent. Control: sham operation.

### Correlations Between the Myocardial Perfusion Parameters (A, β, and A × β) and the SDF-1 RT-PCR Results

**Figure 4:**
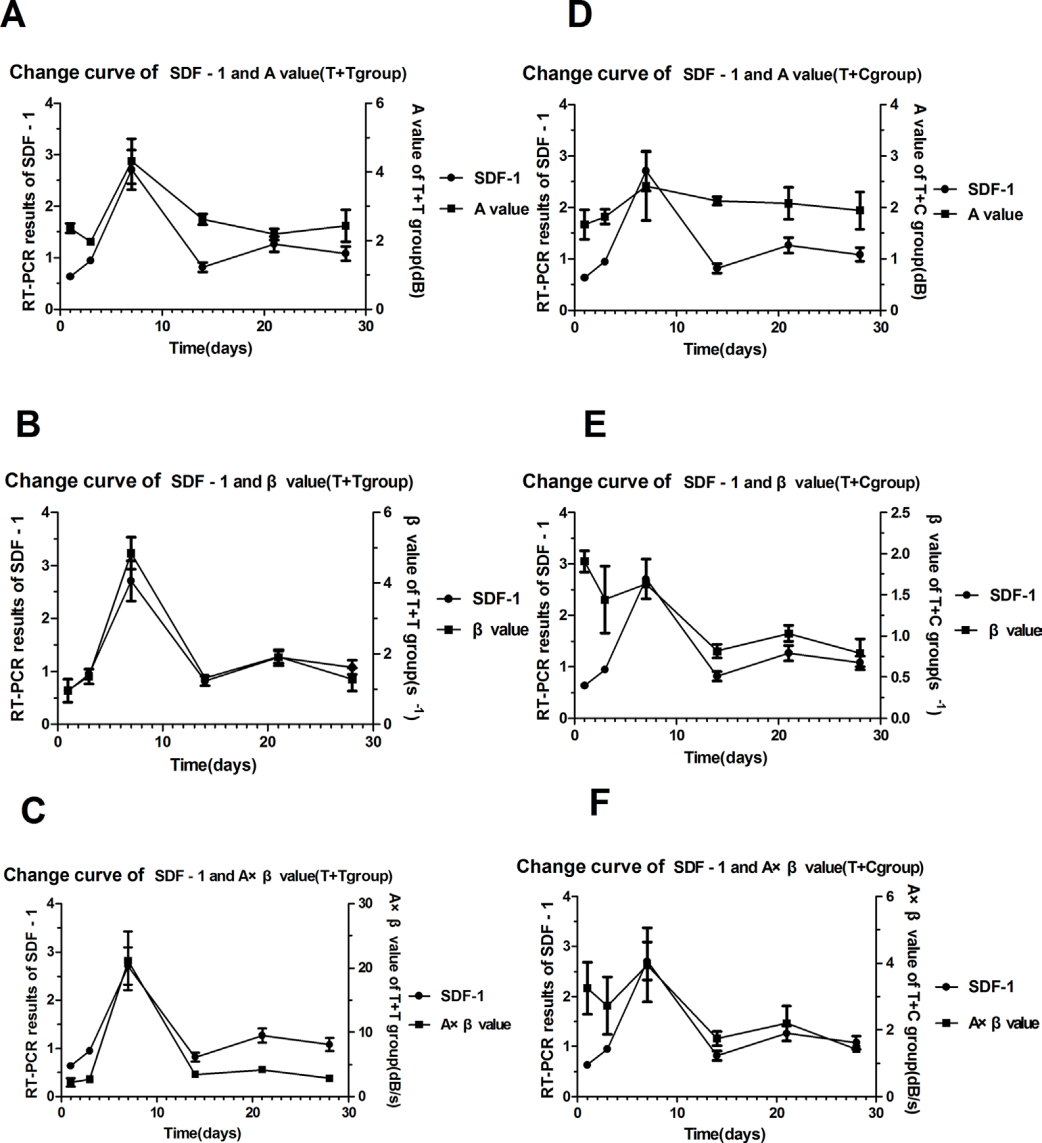
The trend curves for SDF-1 and the myocardial perfusion parameters. **(A) (B) (C)** The trend curves for SDF-1 and the A, β, and A × β values in the T + T group. **(D) (E) (F)** The trend curves for SDF-1 and the A, β, and A × β values in the T + C group.

**Figure 5:**
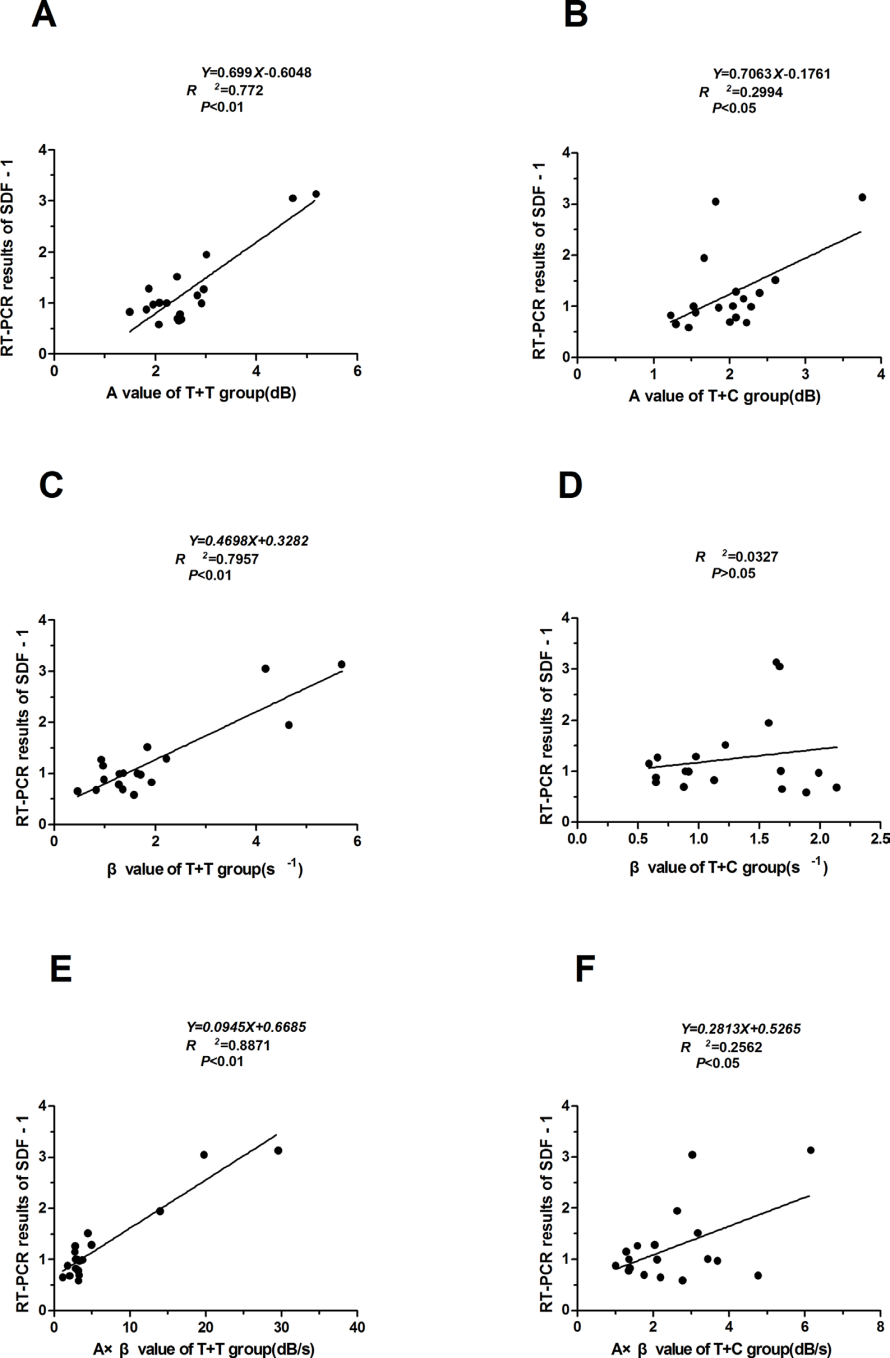
Correlations between the myocardial perfusion parameters (A, β, and A × β) and the SDF-1 RT-PCR results. **(A) (B) (C)** The A, β, and A× β values were correlated with SDF-1 in the T + T group (r = 0.887, 0.892, and 0.942, respectively; *P* < 0.05 and *P* < 0.01). Regression equations were established for the relationships between the A, β, and A × β values (*X*) and SDF-1 (*Y*). **(D) (E) (F)** The A and A × β values were correlated with SDF-1 in the T + C group (r = 0.547 and 0.506, respectively; *P* < 0.05). Regression equations were established for the relationships between the A and A × β values (*X*) and SDF-1 (*Y*). T + T: targeted microbubble ultrasound contrast agent. T + C: nontargeted control microbubbles.

The correlations between the myocardial perfusion parameters (A, β, and A × β) and the SDF-1 RT-PCR results were analyzed, and the regression equations were established. The A and A × β values were correlated with SDF-1 in the T + C group (*r* = 0.547 and 0.506, respectively; *P* < 0.05). The A, β, and A × β values were correlated with SDF-1 in the T + T group (*r* = 0.887, 0.892, and 0.942, respectively; *P* < 0.05 and *P* < 0.01) (**Table 3**). Regression equations were established for the relationships between the A, β, and A × β values (X) and SDF-1 (Y): *Y* = 0.699*X* − 0.6048, *Y* = 0.4698*X* + 0.3282, and *Y* = 0.0945*X* + 0.6685 (*R* ^2^ = 0.772, 0.7957, and 0.8871, respectively; *P* < 0.05 and P < 0.01) (**Figures 4** and **5**).

**Table 3:** Correlations between the myocardial perfusion parameters and SDF-1 mRNA expression.

Group	A(dB/s)		β(澜s^−1^)		A × β(dB/s)	
	*r*	*P*	*r*	*P*	*r*	*P*
**T+C**	0.547	0.019	0.181	0.473	0.506	0.032
**T+T**	0.887	0.000	0.892	0.000	0.942	0.000

The correlations between the myocardial perfusion parameters (A, β, and A × β) and the SDF-1 RT-PCR results. The A and A × β values were correlated with SDF-1 in the T + C group (r = 0.547 and 0.506, respectively; P < 0.05). The A, β, and A × β values were correlated with SDF-1 in the T + T group (r = 0.887, 0.892, and 0.942, respectively; P < 0.05 and P < 0.01).T + T: targeted microbubbles ultrasound contrast agent.T + C: normal ultrasound contrast agent.

### Reproducibility

**Figure 6:**
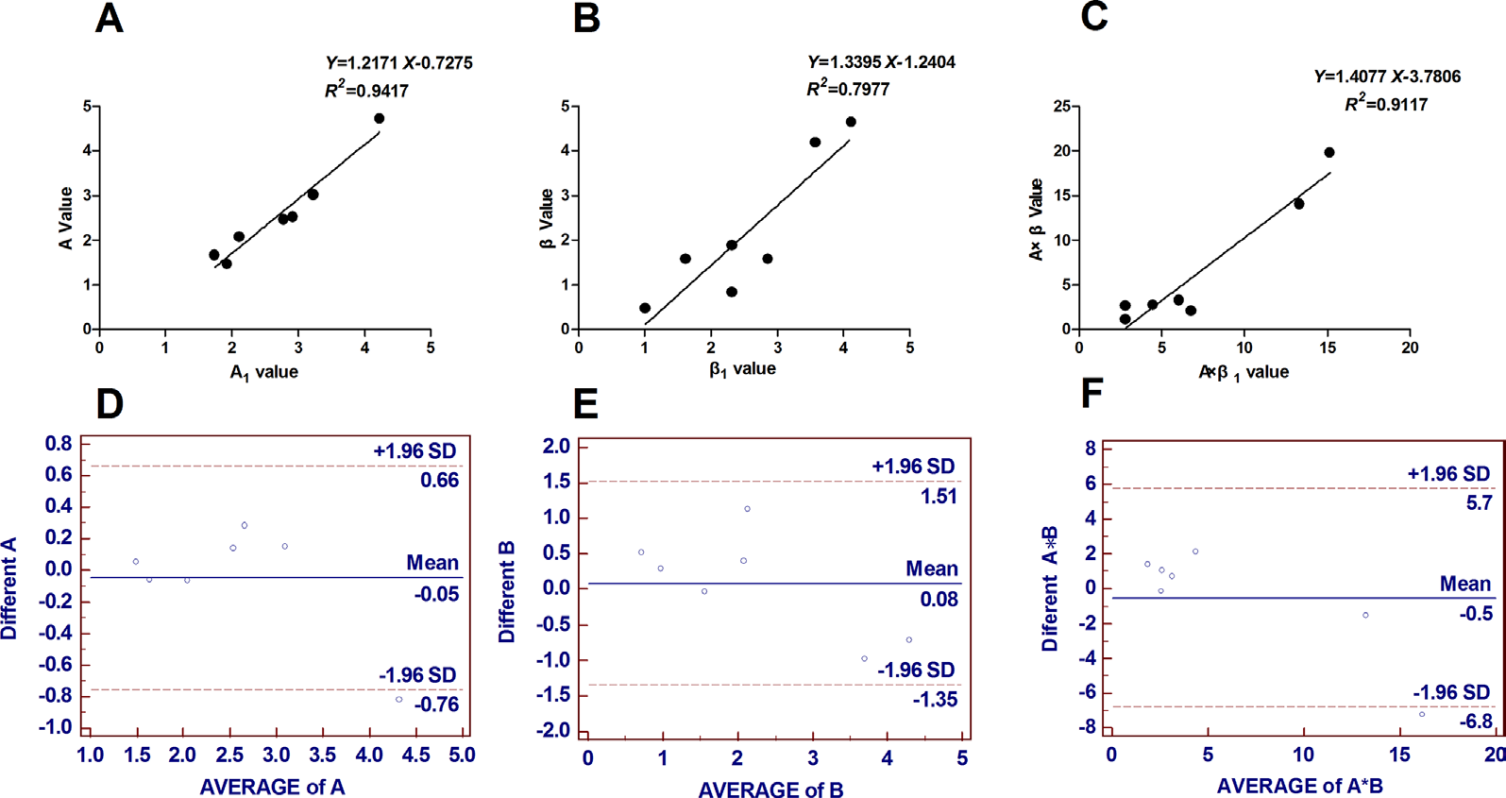
Reproducibility analysis: seven cases were randomly selected to assess intraobserver and interobserver agreement. **(A) (B) (C)** Intraobserver reproducibility results expressed as Pearson correlation coefficients. **(D) (E) (F)** Interobserver reproducibility results obtained from Bland–Altman analyses. Bland–Altman analyses revealed the limits of agreement for A, β and A × β to be −0.76% to 0.66%, −1.35% to 1.51%, and −6.8% to 5.7%, respectively. B = β, A*B = A × β.

The intraobserver and interobserver reproducibility results for all global strain continuous variables are presented in **Figure 6**. Bland–Altman analyses revealed the limits of agreement for A, β, and A × β to be −0.76% in 0.66%, −1.35% in 1.51%, and −6.8% in 5.7%, respectively.

## Discussion

SDF-1 plays an important role in the healing of myocardial injury and promotes stem cell mobilization, migration, proliferation, survival, and differentiation. SDF-1 is expressed at different levels at different time points after myocardial infarction. Numerous studies have confirmed that SDF-1 is an important component of the microenvironments into which stem cells are transplanted. SDF-1 is also a potent chemotactic signal (Liekens et al., 2010; Marcello, 2010; Zaruba and Franz, 2010; Tang et al., 2011). Local SDF-1 expression near the site of myocardial infarction is closely related to the effects of stem cell transplantation. Determining prognosis for myocardial infarction requires recording of the time at which SDF-1 expression peaks (Huber et al., 2011; Wang et al., 2012; Schuh et al., 2014). As shown here, SDF-1 expression in the infarcted myocardial tissues were increased at various time points after AMI. The expression peaked at 1 week and then decreased. Studies investigating variations in the expression of SDF-1 after myocardial infarction have yielded differing results. Munz et al. (2011) found that SDF-1 expression peaks at 1 week after myocardial infarction, which is consistent with the current results. However, Boyle et al. (2011) observed a temporary decline in SDF-1 expression shortly after myocardial infarction. Askari et al., (2003) detected SDF-1 expression at 1 h and 1 day after myocardial infarction but not at 1 or 4 weeks after myocardial infarction or in a sham group. Abbott et al. (2004) reported that SDF-1 expression increases 1 day after myocardial infarction, peaks at 2 days, and remains relatively high during the 2 weeks following myocardial infarction, finally returning to normal levels by 3 weeks after infarction. Ma et al. (2005) observed a peak in SDF-1 expression 1 day after myocardial infarction, followed by a gradual decrease and a return to normal levels after 2 weeks. The discrepancies in these results may be due to the differences in the experimental animals, sampling methods, and detection methods used.

The results of this study indicated that changes in the periodic variation of the SDF-1 content were more prominent in the T + T group than those in the T + C group. The myocardial perfusion parameters (A, β, and A × β), which reflect the perfusion of myocardial microcirculation, exhibited a correlation with the relative quantitative SDF-1 PCR results. These results demonstrated that the targeted ultrasound contrast agent was more effective than the general agent for the noninvasive *in vivo* evaluation of the dynamic changes in SDF-1, and this improved performance may be due to the targeted microbubbles containing SDF-1 monoclonal antibody. These microbubbles can specifically bind to SDF-1, causing the targeted myocardial contrast agent to exhibit stronger specific affinity for the infarcted myocardia. The A × β value of the T + T group and the relative quantitative SDF-1 PCR results were used to establish the regression equations. The results indicated that the A × β value was more accurate than the other indices in the noninvasive *in vivo* evaluation of dynamic changes in SDF-1. The A × β value is expected to become the most commonly used quantitative parameter for evaluating changes in SDF-1 expression over time and facilitates the quantitative analysis of SDF-1 levels after myocardial infarction.

## Conclusions

In summary, ultrasound molecular imaging using microbubbles targeting SDF-1 was found to facilitate the evaluation of changes in SDF-1 expression *in vivo* over time after AMI. The primary limitation of the study was the relatively small number of animals used.

## Ethics Statement

The investigation complied with the Guide for the Care and Use of Laboratory Animals published by the US National Institutes of Health (NIH Publication No. 85-23, revised 1985) and the ARRIVE (Animal Research: Reporting *In Vivo* Experiments) guidelines. All animal procedures were approved by the Care of Experimental Animals Committee of the First Affiliated Hospital of Xinjiang Medical University, China. All animals were housed in an environmentally controlled facility with water *ad libitum*, and received humane care in compliance with institutional guidelines.

## Author Contributions

MW and RH were mainly involved in the experiment and were responsible for writing the manuscript. YY and LX participated in the experiments and data analysis. YM was mainly responsible for the experimental design and peer review.

## Funding

This study was supported by a grant from the National Natural Science Foundation of China (30800480). Parts of this manuscript have previously been presented as abstract at the conference of American College of Cardiology (Yuming et al.).

## Conflict of Interest Statement

The authors declare that the research was conducted in the absence of any commercial or financial relationships that could be construed as a potential conflict of interest.
