# Supplementary material for: In Vivo Ultrasound Molecular Imaging of SDF-1 Expression in a Swine Model of Acute Myocardial Infarction
Source: Front Pharmacol. 2019 Aug 21;10:899. doi: 10.3389/fphar.2019.00899 (PMC6712163; doi:10.3389/fphar.2019.00899)

**Gross specimen of myocardial infarction area**

| Group | n | $\overline{X}$±*s*(cm^2^) | F | *P* |
| --- | --- | --- | --- | --- |
| 1d | 3 | 3.18±0.20^*^ | 0.29 | 0.91 |
| 3d | 3 | 3.04±0.63^*^ |  |  |
| 1w | 3 | 2.79±0.49^*^ |  |  |
| 2w | 3 | 3.15±0.53^*^ |  |  |
| 3w | 3 | 3.14±0.39^*^ |  |  |
| 4w | 3 | 3.17±0.55^*^ |  |  |

Data are presented as the mean ± standard deviation. The differences in the experimental groups (different time points after acute myocardial infarction) were not statistically significant (^*^*P*>0.05).


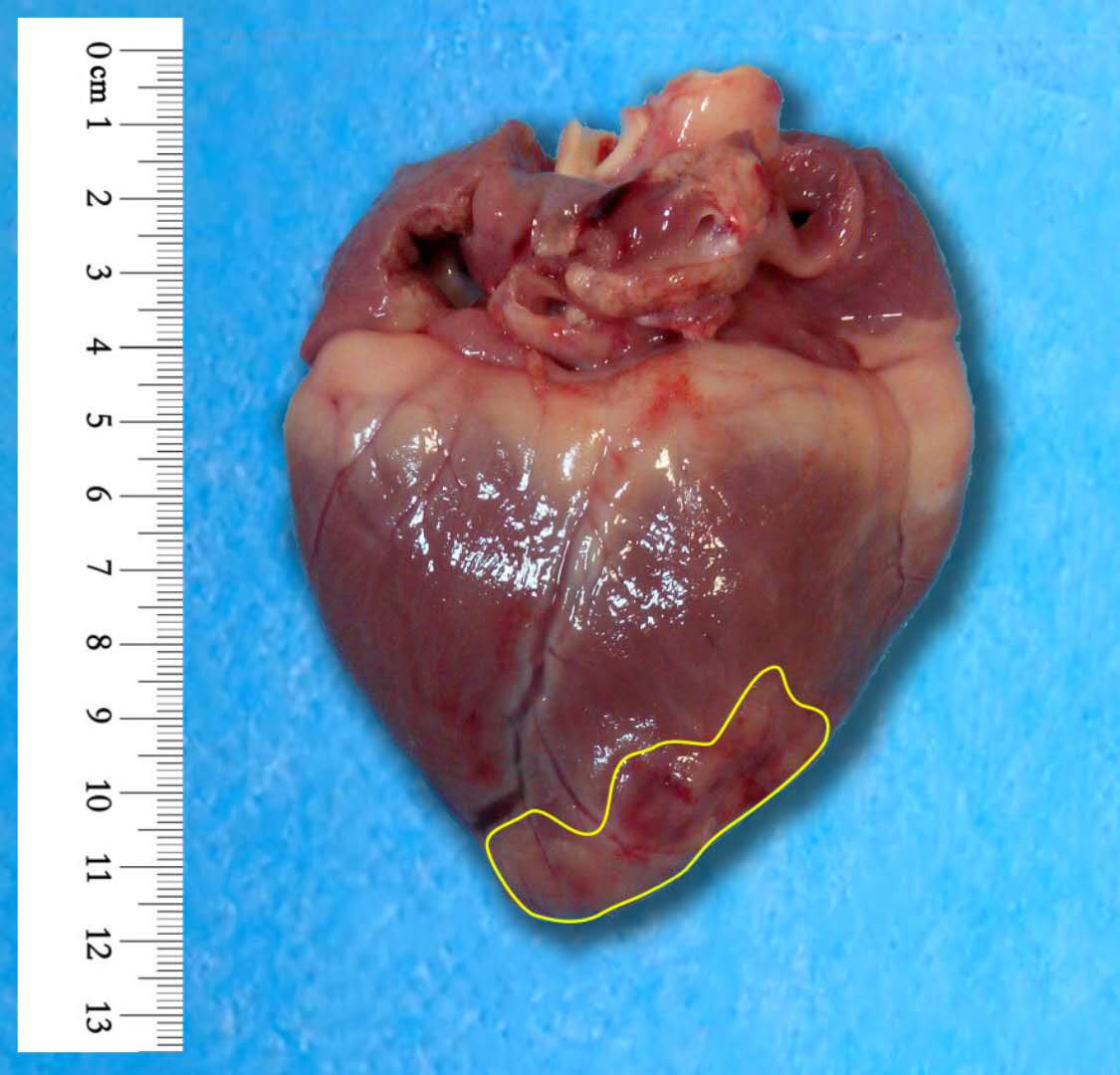

Supplement: Supplementary file 1 [file Table_1.doc]
