# Supplementary material for: In Vivo Ultrasound Molecular Imaging of SDF-1 Expression in a Swine Model of Acute Myocardial Infarction
Source: Front Pharmacol. 2019 Aug 21;10:899. doi: 10.3389/fphar.2019.00899 (PMC6712163; doi:10.3389/fphar.2019.00899)

**Myocardial perfusion score of ECT**

| Group | n | $\overline{X}$±*s* | F | *P* |
| --- | --- | --- | --- | --- |
| 1d | 3 | 15.33±2.52^*^ | 0.15 | 0.98 |
| 3d | 3 | 14.00±2.00^*^ |  |  |
| 1w | 3 | 14.67±1.53^*^ |  |  |
| 2w | 3 | 15.00±3.61^*^ |  |  |
| 3w | 3 | 14.67±1.53^*^ |  |  |
| 4w | 3 | 15.33±1.53^*^ |  |  |

Data are presented as the mean ± standard deviation. The differences in the experimental groups (different time points after acute myocardial infarction) were not statistically significant (^*^*P*>0.05).


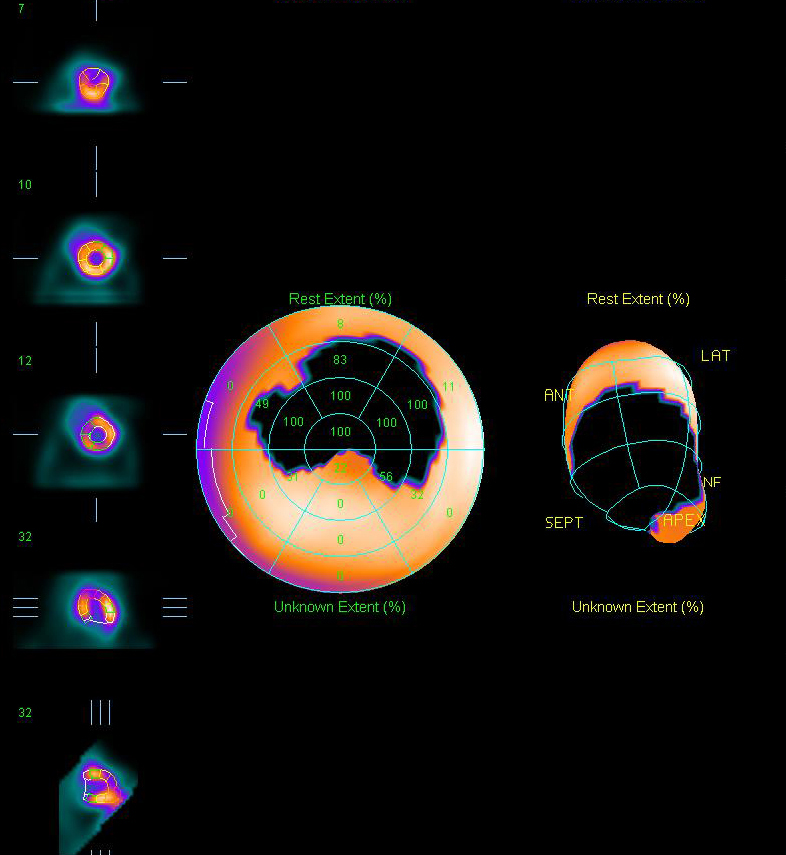

Supplement: Supplementary file 2 [file Table_2.doc]
