# Supplementary material for: In Vivo Ultrasound Molecular Imaging of SDF-1 Expression in a Swine Model of Acute Myocardial Infarction
Source: Front Pharmacol. 2019 Aug 21;10:899. doi: 10.3389/fphar.2019.00899 (PMC6712163; doi:10.3389/fphar.2019.00899)
Supplement: Supplementary file 3 [file Table_3.doc]

**Myocardial enzymes within 24 hours after acute myocardial infarction**

|  | LDH(U/L) | CK(IU/L) | CK-MB(U/L) | hs-cTnT(ng/ml) |
| --- | --- | --- | --- | --- |
| 24h-a | 1322.00 | 16819.00 | 285.10 | 0.191 |
| 24h-b | 1319.50 | 12119.10 | 205.20 | 0.307 |
| 24h-c | 1222.19 | 18205.82 | 255.86 | 0.258 |
| 72h-a | 1527.15 | 18737.19 | 304.83 | 0.319 |
| 72h-b | 2495.12 | 8821.42 | 582.03 | 0.216 |
| 72h-c | 2041.24 | 6625.19 | 442.09 | 0.228 |
| 1w-a | 1264.89 | 5568.55 | 185.58 | 0.335 |
| 1w-b | 1552.2 | 9919.62 | 321.04 | 0.269 |
| 1w-c | 2112.09 | 1216.21 | 455.12 | 0.298 |
| 2w-a | 1752.53 | 11635.58 | 431.70 | 0.353 |
| 2w-b | 2168.75 | 13609.26 | 525.14 | 0.203 |
| 2w-c | 2231.19 | 1189.25 | 269.19 | 0.199 |
| 3w-a | 5494.03 | 1068.75 | 2203.01 | 0.248 |
| 3w-b | 2737.83 | 7598.26 | 898.68 | 0.269 |
| 3w-c | 1692.41 | 9928.19 | 369.56 | 0.303 |
| 4w-a | 1452.32 | 18871.32 | 316.35 | 0.228 |
| 4w-b | 1889.24 | 88203.62 | 521.45 | 0.286 |
| 4w-c | 2015.26 | 11251.24 | 421.68 | 0.295 |
